# Supplementary material for: Modelling daily weight variation in honey bee hives
Source: PLoS Comput Biol. 2023 Mar 1;19(3):e1010880. doi: 10.1371/journal.pcbi.1010880 (PMC9977058; doi:10.1371/journal.pcbi.1010880)
Supplement: S1 Fig — (a) The time spent inside, τd, and outside the hive during the day, τa1 is compared with reported intervals of time inside the hive taken by the forager bees between trips, and an average of time outside the hive per flight. Then, a valid interval is selected considering previous reports. (b) The maximum reported food load collected per trip (crop load) is 0.077 g. The shaded area represents a region where an acceptable crop load is obtained. (c) Representation of two characteristic parameters, the rate of gained weight and the intersection with t = 0, as Nmax decreases B and α become unstable. (d) Error calculated as the sum of the squared difference between the model and the real data. The shaded area represents a region where an acceptable error occurs based on bootstrapping simulation. (PDF) [file pcbi.1010880.s001.pdf]

Supporting Information

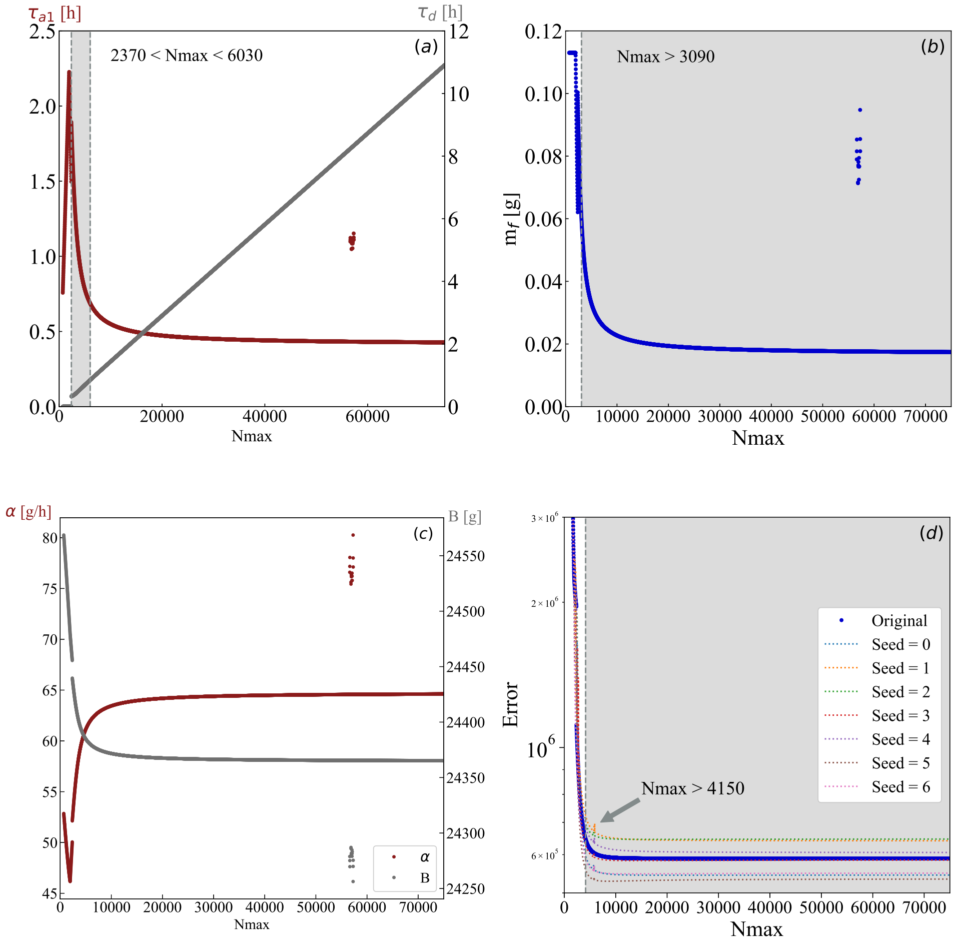

S 1 Fig. Multiple solutions are obtained from the Hive 6 on 2018-4-7, and then reduced to a confidence interval. *(a) The time spent inside,* τ_d*, and outside the hive during the day,* τ_{a_1} *is compared with reported intervals of time inside the hive taking by the forager bees between trips, and an average of time outside the hive per flying. Then, a valid interval is selected considering previous reports. (b) The maximum reported food load collected per trip (crop load) is* 0.077 *g. The shaded area represents a region where an acceptable crop load is obtained. (c) Representation of two characteristic parameters, the rate of gained weight and the intersection with* $t = 0$*, as* N_{max} *decreases* B *and* α *become unstable. (d) Error calculated as the sum of the squared difference between the model and the real data. The shaded area represents a region where an acceptable error occurs based on bootstrapping simulation.*
