# Supplementary material for: Modelling daily weight variation in honey bee hives
Source: PLoS Comput Biol. 2023 Mar 1;19(3):e1010880. doi: 10.1371/journal.pcbi.1010880 (PMC9977058; doi:10.1371/journal.pcbi.1010880)
Supplement: S2 Fig — The outliers detected in red were removed before the data analysis. (PDF) [file pcbi.1010880.s002.pdf]

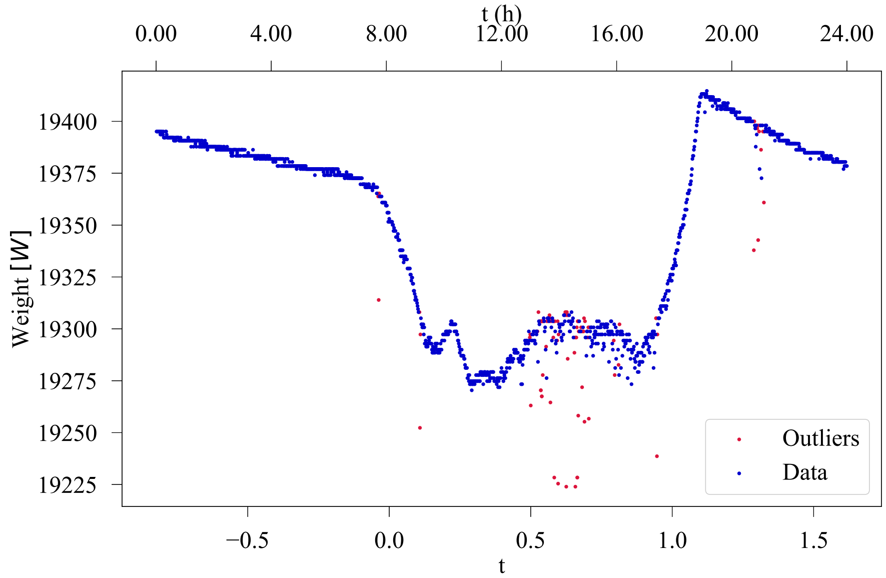

S 2 Fig. Example of outlier detection for the Hive 10 on 2018-4-7. *The outliers detected are in red color and were removed before the data analysis.*
