# Supplementary material for: Modelling daily weight variation in honey bee hives
Source: PLoS Comput Biol. 2023 Mar 1;19(3):e1010880. doi: 10.1371/journal.pcbi.1010880 (PMC9977058; doi:10.1371/journal.pcbi.1010880)
Supplement: S3 Fig — These fitting parameters are: Nmax(bees), W0(kg), m(g), a1(h−1), a2(h−1), d(h−1), l(g/h) with t0 = 8.4 h and t1 = 17.93 h after bootstrapping process of ‘Hive 6’ during the 2018–4-7. (PDF) [file pcbi.1010880.s003.pdf]

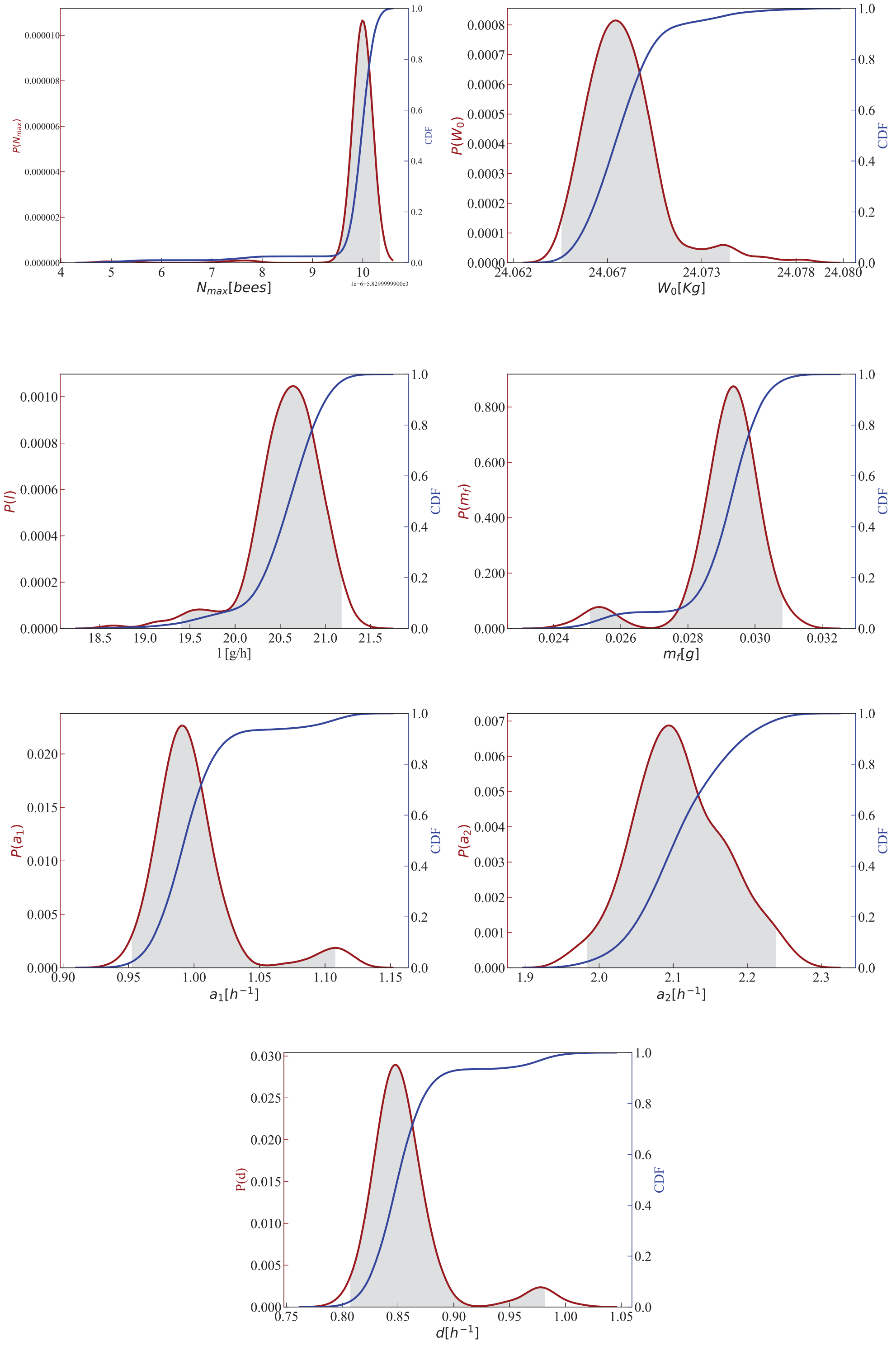

S 3 Fig. Probability Density Function for the parameters displayed at Table 1 after the bootstrapping process of the Hive 6 on 2018-4-7. *These fitting parameters are:* N_{max}*(bees),* W_0*(kg), m(g),* $a_1(h^{-1})$*,* $a_2(h^{-1})$*,* $d(h^{-1})$*, l(g/h) with* $t_0 = 8.4$ *h and* $t_1 = 17.93$ *h after bootstrapping process of 'Hive 6' during the 2018-4-7.*
